# Early activation of pro-fibrotic WNT5A in sepsis-induced acute lung injury

**DOI:** 10.1186/s13054-014-0568-z

**Published:** 2014-10-21

**Authors:** Jesús Villar, Nuria E Cabrera-Benítez, Angela Ramos-Nuez, Carlos Flores, Sonia García-Hernández, Francisco Valladares, Josefina López-Aguilar, Lluís Blanch, Arthur S Slutsky

**Affiliations:** CIBER de Enfermedades Respiratorias, Instituto de Salud Carlos III, Madrid, Spain; Multidisciplinary Organ Dysfunction Evaluation Research Network, Research Unit, Hospital Universitario Dr. Negrin, Las Palmas de Gran Canaria, Spain; Keenan Research Center for Biomedical Science, Li Ka Shing Knowledge Institute, St. Michael’s Hospital, Toronto, Canada; Research Unit, Hospital Universitario NS de Candelaria, Santa Cruz de Tenerife, Spain; Department of Anatomy, Pathology & Histology, Medical School University of La Laguna and Hospital Universitario de Canarias, La Laguna, Tenerife Spain; Critical Care Center, Corporació Sanitaria Parc Taulí, Sabadell, Barcelona Spain; Interdepartmental Division of Critical Care Medicine, University of Toronto, Toronto, ON Canada

## Abstract

**Introduction:**

The mechanisms of lung repair and fibrosis in the acute respiratory distress syndrome (ARDS) are poorly known. Since the role of WNT/β-catenin signaling appears to be central to lung healing and fibrosis, we hypothesized that this pathway is activated very early in the lungs after sepsis.

**Methods:**

We tested our hypothesis using a three-step experimental design: (1) *in vitro* lung cell injury model with human bronchial epithelial BEAS-2B and lung fibroblasts (MRC-5) cells exposed to endotoxin for 18 hours; (2) an animal model of sepsis-induced ARDS induced by cecal ligation and perforation, and (3) lung biopsies from patients who died within the first 24 hours of septic ARDS. We examined changes in protein levels of target genes involved in the Wnt pathway, including WNT5A, non-phospho (Ser33/37/Thr41) β-catenin, matrix metalloproteinase-7 (MMP7), cyclin D1, and vascular endothelial growth factor (VEGF) by Western blotting and immunohistochemistry. Finally, we validated the main gene targets of this pathway in experimental animals and human lungs.

**Results:**

Protein levels of WNT5A, non-phospho (Ser33/37/Thr41) β-catenin, total β-catenin, MMP7, cyclin D1, and VEGF increased after endotoxin stimulation in BEAS-2B and MRC-5 cells. Lungs from septic animals and from septic humans demonstrated acute lung inflammation, collagen deposition, and marked increase of WNT5A and MMP7 protein levels.

**Conclusions:**

Our findings suggest that the WNT/β-catenin signaling pathway is activated very early in sepsis-induced ARDS and could play an important role in lung repair and fibrosis. Modulation of this pathway might represent a potential target for treatment for septic and ARDS patients.

**Electronic supplementary material:**

The online version of this article (doi:10.1186/s13054-014-0568-z) contains supplementary material, which is available to authorized users.

## Introduction

Acute respiratory distress syndrome (ARDS) is a severe inflammatory process caused by pulmonary or systemic insults to the lung alveolar-capillary barrier [1-3]. Sepsis is the most common predisposing factor underlying ARDS and is characterized by systemic inflammation in response to circulating microbes or microbial toxins such as lipopolysaccharide (LPS), also termed endotoxin, a component of the cell wall of gram-negative bacteria. Sepsis and sepsis-induced ARDS are common syndromes associated with high morbidity and mortality [1,2,4]. Effective repair of the alveolar epithelium requires proliferation and migration of type-II alveolar epithelial cells, and their differentiation into type-I alveolar cells [1,5]. In addition, lung fibroblast migration and proliferation occur early after lung injury and are necessary for ongoing lung healing [6-8]. Damage to the alveolar epithelium can lead to abnormal repair that culminates in a vigorous fibroblastic response, leading to uncontrolled extracellular matrix deposition and destruction of lung parenchymal architecture [8,9].

The role of β-catenin-mediated wingless integration (Wnt) signaling is proving to be central to mechanisms of lung healing and fibrosis [10,11]. Tissue repair involves re-epithelialization, in which injured cells are replaced by cells of the same type and normal parenchyma may be replaced by connective tissue leading to fibrosis [11]. Königshoff *et al*. [10] showed that WNT ligands induce lung epithelial cell proliferation, fibroblast activation, and collagen synthesis, and is upregulated in a bleomycin-induced lung injury model and also in humans with idiopathic pulmonary fibrosis. Wnt binding to cognate Frizzled receptors results in cytosolic accumulation of β-catenin, which then translocates to the nucleus and participates in gene transcription [11-13]. Wnt/β-catenin signaling stimulates tissue remodeling and wound closure, or tissue remodeling and destruction through matrix metallopeptidases (MMPs) and other gene products [14]. This activation stimulates many of the pro-inflammatory cytokines participating in inflammation-mediated lung destruction and hyaline membrane formation [12], and induces expression of growth-associated genes such as cyclin D1 and vascular endothelial growth factor (VEGF) [15]. MMP7 (also known as matrilysin) is a target gene of the Wnt signaling pathway found on the surface of lung epithelial cells and is a key regulator of pulmonary fibrosis [16].

In the present study, we examined the hypothesis that the Wnt/β-catenin pathway is activated in the lungs very early after sepsis and plays a role in initiating the lung repair process. To test this hypothesis we used a well-established LPS-induced cell injury model using human lung cells based on the first steps in the development of sepsis and sepsis-induced ARDS [17-21]. Then, we validated the main gene targets of this pathway in a clinically relevant murine model of sepsis-induced ARDS by cecal ligation and perforation (CLP), and in lung biopsies obtained from patients who died within the first 24 h of septic ARDS.

## Materials and methods

### *In vitro* studies

We used healthy human bronchial epithelial BEAS-2B cells (ATCC, Manassas, VA, USA) and human lung MRC-5 fibroblasts. BEAS-2B cells were cultured as previously described [17] in Dulbecco’s modified Eagle’s medium supplemented with 10% FBS and penicillin and streptomycin, at 37°C in a 5% CO_2_, 95% humidified air incubator. MRC-5 cells were obtained from the Department of Microbiology (Hospital Universitario Dr Negrín, Las Palmas, Spain) and cultured in RPMI-1640 medium with 10% FBS under the same experimental conditions. We chose human BEAS-2B and MRC-5 cells as representative lines for studying changes during acute lung injury because these cell lines have been validated previously in experimental models addressing the first steps of sepsis-induced ARDS [18-24]. For all experiments, BEAS-2B and MRC-5 cells were stimulated with 100 ng/mL of LPS obtained from *Escherichia coli* (Sigma-Aldrich, St Louis, MO, USA), a concentration used in previous studies for induction of inflammatory responses [18,21] and validated to study LPS-induced effects [22].

### Inhibition of cell proliferation

BEAS-2B and MRC-5 cells were suspended in 5 × 10^6^ cells/flask and inoculated in 75 cm^2^ flasks. After 24 h, cells were exposed or not, to LPS (100 ng/mL) for 18 h, and then examined and photographed (Olympus Camedia digital camera) under a phase-contrast microscope (Olympus CK-40 F-200, Tokyo, Japan). The effects of LPS on cell growth were assessed using the Sulforhodamine B colorimetric assay (SRB, Sigma-Aldrich) [25] (see Additional file 1 for details).

### Western blotting

Protein levels of WNT5A, total β-catenin, non-phospho (Ser33/37/Thr41) β-catenin, MMP7, cyclin D1, and VEGF were measured by western blotting. For total protein extracts, cells were homogenized in radioimmunoprecipitation assay (RIPA) protein extract buffer, as described previously [26] (see Additional file 1 for further details). Bands were detected by chemiluminescence (Amersham Reagents, GE Healthcare, Fairfield, CN, USA) and blots were measured by Scion Image software package (Scion Corp, Frederick, MD, USA).

### *In vivo* experimental animal model

In an attempt to translate the *in vitro* observations into the disease state of interest (sepsis and ARDS), we performed histological and immunohistochemical examination of lungs from a clinically relevant experimental animal model of sepsis-induced lung injury. The experimental protocol was approved by the Animal Care Committee at the Hospital Universitario Dr Negrin, Las Palmas de Gran Canaria, Spain (CEEBA#003/10), in accordance with the European Commission Directive 2010/63/EU for animal experimentation. This study followed the guidelines, Animal Research: Reporting of in Vivo Experiments (ARRIVE), for reporting animal research [27].

We studied eight healthy male Sprague-Dawley rats weighing 300 to 350 g. After anesthesia with intraperitoneal injection of xylazine and ketamine hydrochloride, animals were randomized to control (sham-sepsis) (n = 3) or sepsis (n = 5). Sepsis was induced by CLP. A detailed description of this experimental model is provided elsewhere [28]. Sham-CLP underwent the same surgical procedures as CLP rats: the cecum was exposed (but not ligated or punctured) and returned to the abdominal cavity, and the abdominal wall was then sutured. Eighteen hours later, control animals and the first three surviving septic animals were anesthetized and sacrificed. A midline thoracotomy/laparotomy was performed and the heart and lungs were removed *en bloc*. The lungs were isolated from the heart, the trachea was cannulated, and the right lung was fixed by intratracheal instillation of 3 mL of 10% formalin and floated in 10% formalin for a week. Lungs were serially sliced from apex to base and embedded in paraffin, cut (3-μm thickness sections) and stained with hematoxylin and eosin for microscope observation. Two pathologists (FV, SGH) were blinded to the sample identity. Three random sections from each animal were examined with particular reference to alveolar and interstitial damage defined by the presence of pulmonary edema, inflammatory cell infiltration, vascular congestion, and fibrosis. Slides were viewed using a Nikon Optiphot 2 microscope and photographed in a Nikon Digital Sight DS-5 M camera (Tokyo, Japan) at × 200 magnification.

We also used the Sirius-red staining technique [29] for assessment of collagen content, as described elsewhere [30]. We defined fibrosis as the presence of collagen. With this technique, collagen fibers are stained bright red and nuclei/cytoplasm are bright yellow. Slides were viewed with an Olympus (Bx50) microscope and photographed with an Olympus digital camera at × 200 magnification.

### Human lung tissue from autopsies

For translating the *in vitro* and *in vivo* observations into the human disease state of interest (sepsis and ARDS), we performed histological and immunohistochemical examination of human lungs from patients who died very early in their course of severe sepsis. Two pathologists (FV, SGH) analyzed the lungs of 12 patients from the archives of autopsies performed between 2007 and 2012 at the Department of Pathology of the University of La Laguna Medical School, Tenerife, Spain. A waiver of ethics was granted by the Ethics Committee for Clinical Research at the Hospital Universitario de Canarias (Tenerife, Spain), as informed consent is systematically obtained from patients’ relatives for both clinical autopsy and potential use of tissue samples in teaching and research purposes. An anonymized summary with clinical relevant information of patients who have had an autopsy is stored in a specific database of the Department of Pathology for further review when necessary. Control lungs were selected from six autopsies in patients who died from diseases without any lung involvement. Septic lungs were selected from autopsies performed in six patients meeting standard criteria for severe sepsis [4] and ARDS [1-3], who did not receive mechanical ventilation and died within the first 24 h of developing severe sepsis. Pathologists were asked to select the autopsies of interest following a strict chronological order, starting with those performed in 2012 and continuing yearly backwards, without any preference or selection bias. After identification of the patients from the postmortem examination, they checked with the institutional database to confirm the clinical diagnosis.

Paraffin blocks of lung tissue collected during autopsy were retrieved from the Department of Pathology archives. In the routine autopsies, three to four fragments of lung parenchyma are obtained. In normal lungs, one fragment of lung tissue was collected from each lobe. The tissue had been fixed in 10% buffered formalin, routinely processed and paraffin embedded. Sections of 3-μm thickness were stained with hematoxylin and eosin and the Sirius-red technique, and evaluated for acute lung injury and collagen content.

### Immunocytochemistry

Immunocytochemical stains were performed by applying a standard avidin-biotin complex technique (see Additional file 1 for further details). To view slides, we used an Olympus BX50 microscope and an Olympus Camedia digital camera at × 400 magnification.

### Statistical analysis

For the statistical power analysis for sample size calculations in both categories of autopsies (diseases with no lung involvement, septic ARDS), we estimated that to detect at least a 2-fold increase in the immunostaining intensity of fibrotic markers (WNT5A, MMP7) in septic lungs compared to the basal intensity in patients without sepsis, we would require six patients in each group, with an alpha of 0.05 and a power greater than 0.80.

Data are expressed as mean ± SD, and were analyzed using Graph Pad Prism software version 5.0. Data are from different experiments and samples within each group. Comparisons involving all experimental cell groups were performed with one-way analysis of variance. We used the Bonferroni correction for multiple comparisons. For western blot experiments, densitometry data of the non-phospho (Ser33/37/Thr41) β-catenin bands were normalized to β-catenin and β-actin (as loading controls), and densitometry of the active form (20 kDa) of MMP7 was normalized to the inactive form (30 kDa) and then normalized to β-actin. Data are from at least three independent experiments. A two-tailed *P*-value <0.05 was considered significant.

## Results

### In vitro studies

LPS decreased the proliferation of MRC-5 and BEAS-2B cells. The maximum effect on cell viability in both cell types was observed at 18 h using 100 ng/mL LPS (data not shown).

### WNT5A and associated proteins

**Figure 1:**
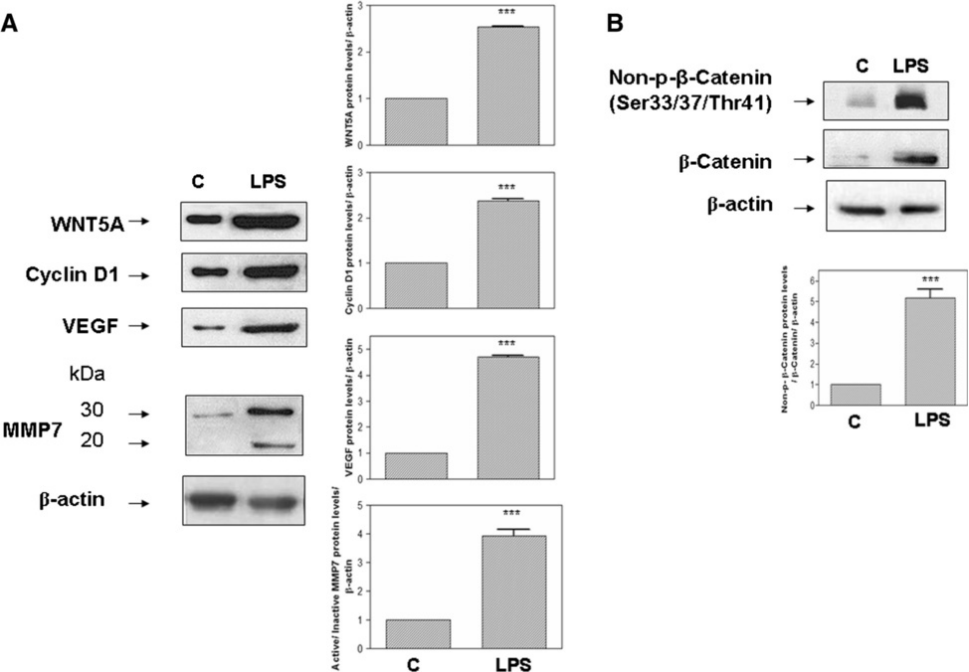
**Activation of WNT5A/β-catenin pathway by** ***E*** **.** ***coli*** **lipopolysaccharide (LPS) in human BEAS-2B cells. (A)** Changes in total WNT5A, cyclin D1, metallopeptidase (MMP)7 and vascular endothelial growth factor (VEGF) proteins (representative blots and mean densitometric values) following LPS stimulation for 18 h. Densitometry analysis of the active form (20 kDa) of MMP7 was normalized to the inactive form (30 kDa) and then normalized to β-actin. **(B)** Changes in non-phosphorylated (Ser33/37/Thr41) β-catenin protein bands were normalized to total β-catenin and β-actin. ***P <0.001 versus control-vehicle **(C)**.

**Figure 2:**
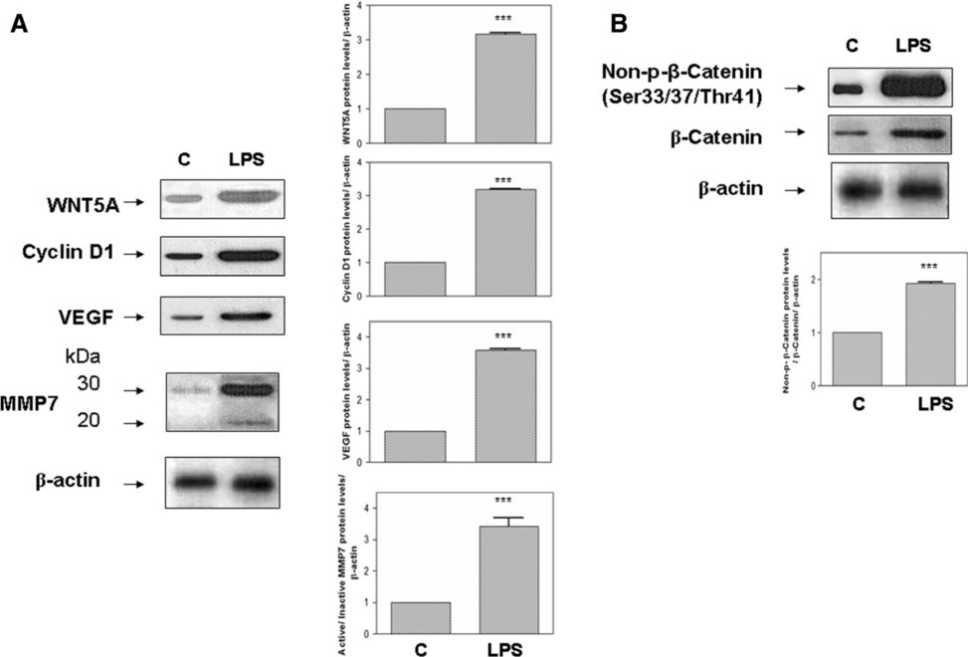
**Representative western blots for WNT5A/β-catenin pathway stimulated by LPS in human MRC-5 cells. (A)** Changes in total WNT5A, cyclin D1, metallopeptidase (MMP)7 and vascular endothelial growth factor (VEGF) proteins following exposure to 100 ng/mL lipopolysaccharide (LPS) stimulation for 18 h. Densitometry analysis of the active form (20 kDa) of MMP7 was normalized to the inactive form (30 kDa) and then normalized to β-actin. **(B)** Changes in non-phospho Ser33/37/Thr41 β-catenin after 18 h of LPS stimulation. Densitometry was performed on at least three different blots per condition and normalized to the respective loading control (β-actin). Protein expression is expressed as fold-change relative to the respective control vehicle **(C)**. ***P <0.001 versus control vehicle **(C)**.

**Figure 3:**
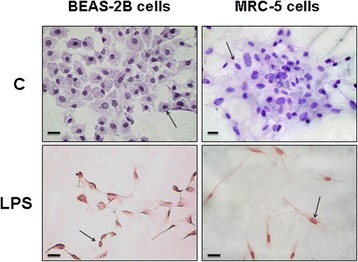
**Non-phospho Ser33/37/Thr41 β-catenin immunolocalization on BEAS-2B and MRC-5 cells stimulated with lipopolysaccharide (LPS).** Red-pink colour indicates positive staining (3-amino-9-ethylcarbazole) for non-phospho Ser33/37/Thr41 β-catenin protein. Non-phospho Ser33/37/Thr41 β-catenin staining was found in nuclei (large arrows) in cells stimulated with LPS but not in control-vehicle cells (C). The images (at × 200 magnification) are representative of experiments performed in triplicate. Scale bars = 20 μm.

WNT5A protein levels were significantly increased in MRC-5 and BEAS-2B cells (*P* <0.001) after LPS exposure (Figures 1A and 2A, respectively). LPS stimulation led to a significant increase in non-phospho (Ser33/37/Thr41) β-catenin (Figures 1B, 2B). The active form of the MMP7 protein was increased in both MRC-5 and BEAS-2B cells stimulated with LPS. LPS treatment also caused increased upregulation of cyclin D1 and VEGF (Figures 1A, 2A). Immunocytochemical staining detected non-phospho Ser33/37/Thr41 β-catenin at the nuclei of MRC-5 and BEAS-2B cells stimulated with LPS (Figure 3).

### Animal model

**Figure 4:**
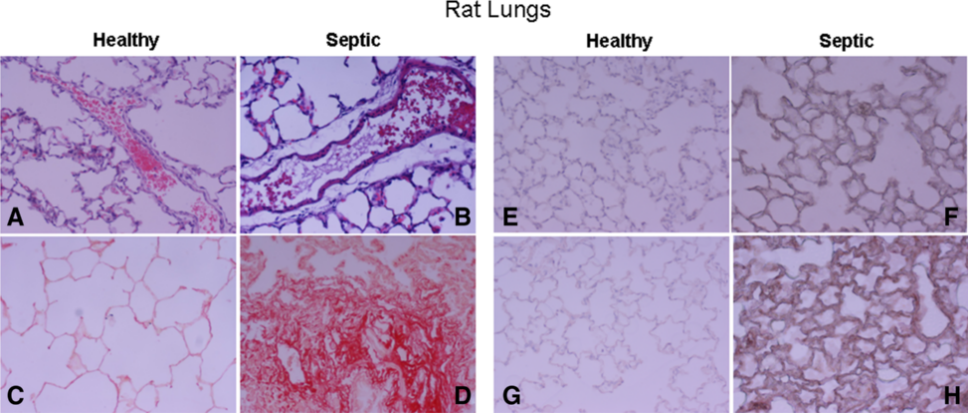
**Representative histological features of healthy and septic rat lungs and immunohistochemical staining for WNT5A and MMP7 activation in healthy and septic rat lungs. (A-D)** Histological features: **(A)** normal lung (healthy) and **(B)** septic lung showing pulmonary infiltrates and perivascular edema; **(C)** normal lung (healthy) and **(D)** septic lung stained with Sirius-red assay for collagen content (× 200 magnification). **(E-** **H)** Red-pink color indicates positive staining for WNT5A **(E,** **F)** and metallopeptidase (MMP)7 **(G,** **H)** and blue/violet indicates nuclei counterstained with hematoxylin. WNT5A and MMP7 were observed in alveolar walls and septa (× 200 magnification).

CLP induced typical signs of disease including lethargy, ruffled fur, generalized weakness, reduced gross motor activity, and weight loss, accordingly with the literature [28]. Three out of five septic animals survived 18 h after CLP, and these animals were studied further. Lungs from septic animals showed acute inflammatory infiltrates, perivascular edema and collagen deposition in the parenchyma (Figure 4, panel B). The Sirius-red staining for collagen was negative in control animals (Figure 4, panel D). Healthy control animals had a basal intensity of WNT5A and MMP7 whereas septic lungs showed strong immunohistochemistry intensity of WNT5A and MMP7 (Figure 4, panels F and H).

### Human lungs from autopsies

**Figure 5:**
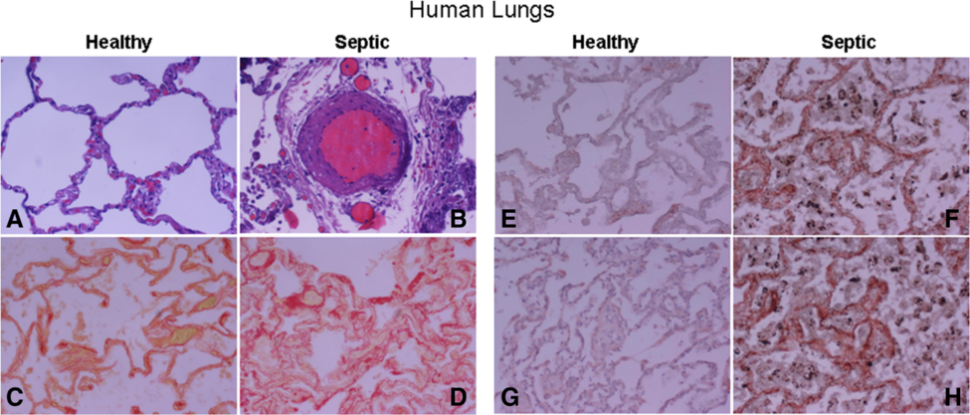
**Representative histological features of healthy and septic human lungs and immunohistochemical staining for WNT5A and MMP7 activation in normal and septic human lungs. (A-D)** Histological features: **(A)** normal lung (healthy) and **(B)** septic lung showing pulmonary infiltrates and perivascular edema; **(C)** normal lung and septic lung **(D)** stained with Sirius-red assay for collagen content (× 200 magnification). **(E-** **H)** WNT5A **(E,** **F)** and metallopeptidase (MMP)7 **(G,** **H)** are shown in red-pink color. Tissues were counterstained with hematoxylin. There was increased immunoreactivity for WNT5A and MMP7 in alveolar walls and septa (× 200 magnification).

Clinical diagnoses of six patients who died with septic ARDS and six control subjects who died from non-pulmonary causes within 24 h of disease onset are presented in Table 1. No relevant findings were found in the lungs from patients who died without lung disease. Lungs from septic patients showed features of diffuse lung damage, manifested by acute inflammatory infiltrates and perivascular edema (Figure 5, panel B). Lungs from septic patients showed high intensity of collagen-rich areas in the parenchyma, providing evidence of the presence of a fibrotic response in the early stages of sepsis-induced lung injury (Figure 5, panel D). Lungs from patients without pulmonary disease had a basal intensity of WNT5A and MMP7 whereas lungs from septic patients showed a strong immunohistochemical intensity of WNT5A and MMP7 (Figure 5, panels F and H)

**Table 1:** **Clinical diagnosis of patients with non-pulmonary diseases and sepsis-induced acute lung injury**

**Main diagnosis**	**Cause/mechanism of death**
**Non-pulmonary diseases**	
Acute myocardial infarction	Ventricular fibrillation
Acute myocardial infarction	Left ventricular rupture
End-stage colon cancer	Myocardial infarction
End-stage liver cirrhosis	Liver failure
Coronary artery disease	Severe cardiac arrhythmias
Epilepsy, Down syndrome	Severe hypoxic encephalopathy
**Sepsis-induced lung injury**	
Acute peritonitis	Septic shock
AIDS	Pneumonia
Pneumonia	Septic shock
Abdominal sepsis	Septic shock
Abdominal sepsis	Septic shock
Pneumonia	Septic shock

## Discussion

We examined the translational impact of the WNT/β-catenin pathway in an LPS-induced human lung cell injury model and validated the main gene targets of this pathway in the lungs of septic experimental animals and in human lungs from autopsies. The major findings of our study are: (1) WNT5A is expressed very early by human airway epithelial cells and lung fibroblasts in response to LPS; (2) upregulation of WNT5A expression and non-phospho Ser33/37/Thr41 β-catenin are associated with upregulation of downstream target genes that are involved in profibrotic transformation of injured tissues, such as MMP7, cyclin D1 and VEGF; and (3) pulmonary fibrosis is induced very early during sepsis-induced ARDS, both experimentally and clinically. These findings suggest that WNT5A and β-catenin contribute very early to repair the damage to lung tissue and may play a role in restructuring lung architecture during sepsis-induced ARDS.

We selected BEAS-2B and MRC-5 cell lines as representative human airway epithelial cells and lung fibroblasts because these cells have been implicated in the pathogenesis of sepsis-induced ARDS [18-24] and subsequent fibrosis [31]. These cell models provide a powerful translational *in vitro* approach for recapitulating human ARDS. LPS-treated human BEAS-2B cells are an accepted and validated *in vitro* cell injury model of the acute lung inflammatory response based on the first steps in the development of sepsis and sepsis-induced ARDS [18]. Lung airway epithelial cells and fibroblasts generate various immune effectors such as cytokines, chemokines, and several peptides in response to inflammatory stimuli [23,32], which control lung inflammation, lung injury and lung repair [9,12,31,33]. We selected *E. coli* LPS because it has been used in most endotoxin-induced lung injury models [21,34] and LPS is a key pathogen recognition molecule for sepsis [33,34]. Because previous *in vitro* studies using LPS-stimulated airway epithelial cells and fibroblasts focused on activation of pro-inflammatory mediators and increased cytokine release [20,35,36], we examined the modulation of WNT5A, β-catenin, MMP7, cyclin D1 and VEGF molecules that contribute to lung repair and fibrosis [12,16,37].

We extended our *in vitro* findings by confirming that collagen synthesis and the main target gene products of this pathway (WNT5A, MMP7) increased in a clinically relevant model of sepsis-induced lung injury and in lungs from patients who died with severe sepsis and ARDS. We used CLP as a clinically relevant and well characterized animal model to explore the fibrotic transformation in the lungs during the first 24 h of sepsis. CLP induced a reproducible and consistent septic and sepsis-induced ARDS condition in accordance with previous studies [17,28]. Histopathological features of CLP-induced ARDS in animals included atelectasis, pulmonary edema, and acute inflammatory infiltrates. Lung tissue damage is observed in 90% of patients dying from sepsis [38]. Moreover, lung cells can activate mechanisms for initiating tissue repair, a process which involves re-epithelialization; injured cells are replaced by cells of the same type, but in some cases, normal parenchyma is replaced by connective tissue leading to fibrosis [11]. There is evidence of fibrotic changes in the earliest stages of ARDS [26,39,40]. β-catenin signaling stimulates tissue remodeling, cell migration, and wound closure through MMPs, but if the process is uncontrolled, it can drive tissue destruction through MMPs and other mediators [11]. Wnt ligands induce lung epithelial cell proliferation, fibroblast activation and collagen synthesis [16]. Collagen and other matrix extracellular molecules are the main components of the extracellular matrix, and MMP7 is a key mediator of pulmonary fibrosis [16].

Several *Wnt* genes are expressed in the developing and adult lung. Of these, *Wnt5a* and *Wnt7b* are expressed at high levels in the airway epithelium [14]. We chose to examine the modulation of WNT5A because it has been implicated in several pulmonary disorders [11] and has not been studied in the context of sepsis and LPS-induced ARDS. In our study, WNT5A was detected with moderate intensity in alveolar walls and septa in the lungs of CLP rats and in the lungs of humans who died with early septic ARDS. Blumenthal *et al*. [41] reported that the expression of WNT5A required Toll-like receptor signaling and NF-κB activation. In a previous report by our group, and using the same epithelial cell injury model as in the present study, we showed that LPS modulated the NF-κB activation through the Toll-like receptor signaling [22]. The fact that β-catenin is rapidly upregulated in our epithelial/fibroblast cell injury model suggests that the WNT/β-catenin pathway could be continuously stimulated during ARDS and it could be a mechanism for perpetuating lung injury or for initiating lung repair. Thus, the activation of Wnt signaling after sepsis-induced ARDS likely represents a regenerative signal of the damaged epithelium [42]. Using expression microarrays, Vuga *et al*. [43] showed that WNT5A was significantly increased in fibroblasts isolated from lung tissues of patients with lung fibrosis compared with fibroblasts from normal lung tissues. They also reported increased cell proliferation when normal lung fibroblasts were treated with WNT5A.

Our findings parallel those of Chilosi *et al*. [44] who found aberrant WNT/β-catenin pathway activation in lungs from patients with idiopathic pulmonary fibrosis, suggesting that this pathway could be responsible for dysfunctional lung repair processes leading to severe and irreversible pulmonary remodeling. This is a relevant translational finding because the development of pulmonary fibrosis has been found to have a direct correlation with severity of lung injury and mortality in ARDS patients [45]. The cell cycle regulatory molecule cyclin D1 gene is one of the target genes for the Wnt/β-catenin signaling pathway, and VEGF is required for maintenance of adult lung alveolar structures. Any tissue repair involves coordinated cellular infiltration together with extracellular matrix deposition and re-epithelialization. Proteolytic degradation of the extracellular matrix requires MMPs which are regulated by Wnt signaling. It is uncertain why ARDS resolution involves fibrosis in some patients but not in others. Using western blot analysis of Wnt target gene products cyclin D1 and MMP7, Königshoff *et al*. [16] demonstrated increased functional Wnt/β-catenin signaling in pulmonary fibrosis compared with patients without pulmonary fibrosis. Zuo *et al*. [46] analyzed samples from patients with pulmonary fibrosis using microarray technology and found that *Mmp7* was the most upregulated gene, a finding that was confirmed by immunohistochemistry. The increased expression of cyclin D1, VEGF, and MMP7 in our study supports the importance of Wnt signaling in perpetuating lung inflammation and provides insights into the early development of a pro-fibrotic response during sepsis-induced ARDS. A greater understanding of modulators of WNT expression and the effects of WNT proteins in similar models will be paramount for clarifying the role of this pathway in lung inflammation and repair.

Our study does have some limitations. First, although the animal model used in the present investigation was CLP, we have examined autopsies from patients with different types of septic ARDS. However, there are no data suggesting that there is anything specific about pulmonary versus non-pulmonary insults in terms of different pulmonary fibrotic responses during severe sepsis. In an acid aspiration lung injury model, we found a similar fibrotic transformation as in our septic model [26]. A recent study [40] has shown that pulmonary fibrosis represents an early pathologic response in patients with ARDS, independent of the pulmonary or extrapulmonary nature of its cause. Second, we did not explore the effects of inhibitors of the Wnt pathway to irrefutably demonstrate that activation of Wnt pathway in the lung by a septic insult is responsible for the upregulation of downstream target genes (such as MMP7, cyclin D1, VEGF) that are involved in the pro-fibrotic transformation of injured tissues. However, studies by other investigators on selective inhibition of the Wnt/β-catenin signaling pathway [44,47,48] have indicated that the WNT/β-catenin pathway is a target for anti-inflammatory and anti-fibrotic actions.

## Conclusion

In summary, our findings suggest that the WNT/β-catenin pathway may contribute to ongoing lung inflammation and lead to a pro-fibrotic response in the early stages of ARDS. We observed increased expression of WNT5A, cyclin D1, VEGF, and MMP7, all of which are *Wnt* target gene products that play an important role in pulmonary fibrosis. Further studies are needed to fully address unresolved questions regarding the modulation of the Wnt signaling pathway for attenuating lung inflammation and enhancing lung resolution and repair as a preventive or therapeutic approach in the setting of sepsis-induced ARDS.

## Key messages

- The role of Wnt signaling is proving to be central to mechanisms of lung healing and fibrosis
- Wnt/β-catenin pathway is activated in the lungs very early after sepsis and plays a role in initiating the lung repair process
- Modulation of the Wnt/β-catenin pathway might represent a potential target for treatment in patients with sepsis and ARDS-induced pulmonary fibrosis

## Additional file

Additional file 1:
**Supplementary information on methods for: (i) morphological analysis and inhibition of cell proliferation, (ii) western blot analysis, and (iii) immunohistochemistry.**

## Abbreviations

ARDS: acute respiratory distress syndrome
CLP: cecal ligation and puncture
FBS: fetal bovine serum
LPS: lipopolysaccharide
MMP: metallopeptidase
MMP7: metallopeptidase (or metalloproteinase) 7 (also known as matrilysin)
VEGF: vascular endothelial growth factor
WNT5A: wingless-type integration site family, member 5A
