# Supplementary material for: Early activation of pro-fibrotic WNT5A in sepsis-induced acute lung injury
Source: Crit Care. 2014 Oct 21;18(5):568. doi: 10.1186/s13054-014-0568-z (PMC4220065; doi:10.1186/s13054-014-0568-z)
Supplement: Additional file 1: — Supplementary information on methods for: (i) morphological analysis and inhibition of cell proliferation, (ii) western blot analysis, and (iii) immunohistochemistry. [file 13054_2014_568_MOESM1_ESM.doc]

***-ADDITIONAL FILE 1-***

**Early activation of pro-fibrotic WNT5A in sepsis-induced acute lung injury**

**^1,2,3*^Jesús Villar**, MD, PhD; **^1,2^Nuria E. Cabrera-Benítez**, PhD; **^1,2^Ángela Ramos-Nuez**, MLT; **^1,4^Carlos Flores**, PhD; **^5^Sonia García-Hernández**, MD, PhD; **^1,5^Francisco Valladares**, PhD; **^1,6^Josefina López-Aguilar**, PhD; **^1,6^Lluís Blanch**, MD, PhD; **^3,7^Arthur S. Slutsky**, MD

jesus.villar54@gmail.com

nuriaecb@gmail.com

shefloanse@hotmail.com

cflores@ull.edu.es

soniagaher76@gmail.com

fvallapa@gmail.com

JLopezA@tauli.cat

lblanch@tauli.cat

SLUTSKYA@smh.ca

*From*

(1) CIBER de Enfermedades Respiratorias, Instituto de Salud Carlos III, Madrid, Spain;

(2) Multidisciplinary Organ Dysfunction Evaluation Research Network, Research Unit, Hospital Universitario Dr. Negrin, Las Palmas de Gran Canaria, Spain;

(3) Keenan Research Center for Biomedical Science, Li Ka Shing Knowledge Institute, St. Michael’s Hospital, Toronto, Canada;

(4) Research Unit, Hospital Universitario NS de Candelaria, Santa Cruz de Tenerife, Spain;

(5) Department of Anatomy, Pathology & Histology, Medical School University of La Laguna, La Laguna, Tenerife, Spain;

(6) Critical Care Center, Corporació Sanitaria Parc Taulí, Sabadell, Barcelona, Spain;

(7) Interdepartmental Division of Critical Care Medicine, University of Toronto, Toronto, Ontario, Canada.

*** Address for correspondence**

Dr. Jesús Villar

Multidisciplinary Organ Dysfunction Evaluation Research Network, Hospital Universitario Dr. Negrin,

Barranco de la Ballena, s/n - 4th floor, South wing. 35010 Las Palmas de Gran Canaria. Spain.

Tel: (+34) 928-449413 Fax: (+34) 928-449813 e-mail: jesus.villar54@gmail.com

**SUPPLEMENTARY METHODS**

**Morphological analysis and inhibition of cell proliferation**

BEAS-2B and MRC-5 cells were suspended in 5x10^6^ cells/flask and inoculated in 75 cm^2^ flasks. After 24 h, cells were then exposed or not to LPS (100 ng/mL) for 18 h, and then and examined and photographed (Olympus Camedia digital camera) under a phase-contrast microscope (Olympus CK-40 F-200).

The effects of LPS on cell growth were investigated using the Sulforhodamine B colorimetric assay (SRB, Sigma-Aldrich) (1). Briefly, BEAS-2B and MRC-5 cells were seeded at a density of 4x10^4^ cells per well in 100 µl of growth medium (DMEM or RPMI-1640, according to cell line) supplemented with 2% FBS in a 96-well plate. After 24 h incubation at 37ºC (in 5% CO_2_, 95% humidified air incubator), 100 µl of LPS dilutions were added to triplicates wells. Cells were incubated at 37ºC (6-18 h) until the control-vehicle cells (no treatment) became confluent. For determination of initial number of cells, an extra plate was set up and processed similarly after 24 h of incubation at 37ºC without treatment. All experimental conditions were fixed with 25 μL ice-cold TCA (50% w/v) and fixed for 60 min at 4ºC, washed five times with tap water, air dried and stained with 0.4% SRB in 1% acid acetic for 1 h. Optical density of each well was measured at 492 nm, using BioTek’s PowerWave XS Absorbance Microplate Reader. Data are expressed as percentage over control-vehicle.

**Western blot analysis**

Protein levels of WNT5A, total β-catenin, non-phospho (Ser33/37/Thr41) β-catenin, MMP7, cyclin D1, and VEGF were measured by Western blotting. For total protein extracts, cells were homogenized in RIPA protein extract buffer, as described previously (2). Homogenized samples (50 µg of protein each) were subjected to 10-12.5% SDS-PAGE under reducing conditions. Proteins were transferred onto PVDF membranes and blocked with 10% skim milk in Tris-buffered saline plus 0.1% Tween 20. Detection of WNT5A (Abcam, Cambridge, UK), total β-catenin, MMP7, cyclin D1, and VEGF (Santa Cruz Biotechnology, Santa Cruz, CA), non-phospho (Ser33/37/Thr41) β-catenin (Cell Signalling Technology, Danvers, MA) were performed in random samples by western blotting using rabbit polyclonal anti-WNT5A, anti-β-catenin, anti-non-phospho (Ser33/37/Thr41) β-catenin, and anti-MMP7 antibodies and a goat anti-rabbit IgG-streptavidin horseradish peroxidase (HRP) as secondary antibody (Santa Cruz Biotechnology); mouse monoclonal antibody anti-cyclin D1 and a rabbit antimouse IgG-HRP as secondary antibody (Dako, Glostrup, Denmark), goat polyclonal anti-VEFG and a donkey anti-goat IgG-HRP as secondary antibody (Santa Cruz Biotechnology). Loading control was assessed with a rabbit anti-β-actin antibody (Cell Signalling). In all cases, bands were detected by chemiluminescence (Amersham Reagents, GE Healthcare, Fairfield, CN) and blots were measured by Scion Image software package (Scion Corp, Frederick, MD).

**Immunocytochemistry**

Immunocytochemical stains were performed by applying a standard avidin-biotin complex technique. Cells were cultured on duplicate chambers of an 8-chamber glass slide (Nunc Lab-Tek II Chamber Slide) in the absence or presence of 100 ng/ml LPS for 18 h. After LPS treatment, cells were fixed in 4% paraformaldehyde in PBS for 20 min, washed twice with PBS and permeabilized with 0.2% Triton X-100 for 10 min and thereafter incubated for 1 h with primary antibodies directed against non-phospho Ser33/37/Thr41 β-catenin. After primary antibody incubation, cells were washed in PBS and incubated for 12 min with a biotin-conjugated secondary antibody (Santa Cruz Biotechnology). After secondary antibody incubation, slides were incubated for 10 min at room temperature with streptavidin-HRP. Staining was visualized using 3-amino-9-ethylcarbazole AEC+/substrate chromogen.

Immunohistochemical stains for WNT5A and MMP7 in samples from rat and human lungs were performed in sections from multiple regions according to a standard avidin-biotin complex technique, as previously described (2) using 3-amino-9-ethylcarbazole as substrate (red-pink color assay). A blue/violet color indicates nuclei counterstained with hematoxylin. We used rabbit polyclonal primary antibodies against WNT5A (Abcam, Cambridge, UK) and MMP7 (Santa Cruz Biotechnology). To view slides, we used an Olympus BX50 microscope and an Olympus Camedia digital camera at x400 magnification.

**SUPPLEMENTARY REFERENCES**

1. Skehan P, Storeng R, Scudiero D, Monks A, McMahon J, Vistica D, Warren JT, Bokesch H, Kenney S, Boyd MR: **New colorimetric cytotoxicity assay for anticancer-drug screening.** *J Natl Cancer Inst* 1990, **82**:1107-1112.
2. Villar J, Cabrera N, Casula M, Flores C, Valladares F, Muros M, Blanch L, Slutsky AS, Kacmarek RM: **Mechanical ventilation modulates Toll-like receptor signaling pathway in a sepsis-induced lung injury model.** *Intensive Care Med* 2010, **36**:1049-1057.
